# The Ophthalmoscope in General Practice

**Published:** 1900-02-10

**Authors:** 


### THE OPHTHALMOSCOPE IN GENERAL PRACTICE.

In his presidential address to the Harveian Society Mr. Henry Juler, among many other good things which he said, took the opportunity of impressing upon his hearers the desirability of keeping up their practical familiarity with the use of the ophthalmoscope. The great charm of ophthalmic practice seemed to him to lie in the precise and definite nature of the work; for it is possible in the majority of cases to give a definite diagnosis, a definite line of treatment, and a definite prognosis. This is undoubtedly due in a large measure to the proper and skilful use of the ophthalmoscope, by aid of which instrument we can, by retinoscopy, find out in a few minutes, not only the quality of an error of refraction, but can estimate its quantity with the greatest accuracy, while by the direct method of ophthalmoscopic examination we can scan the retina, the optic nerve, and the choroid; a few drops of hydro-bromate of homatropine instilled into the palpebral sac half-an-hour beforehand rendering this quite a simple matter. Among other uses of the ophthalmoscope mentioned is the detection of malingerers, and if conscription were to come into force this would be a matter of much importance. Even now (says Mr. Juler) it happens sometimes that people believe, or pretend, that they cannot see. They generally complain of one eye only. Many of them belong to the school-girl type, and are between the ages of 10 and 18 years. An anxious mother states that the girl cannot see with one eye; yet when we examine the pupils we may find them both equal, active to light, and normal in size. By retinoscopy, again, we may find that there is no ametropia such as would cause blindness, and by the ophthalmoscope we may find the fundus absolutely normal. Still the patient declares that with the one eye she cannot see. In such a case Mr. Juler proceeds as follows, it being premised that the room having been darkened for the preceding examination, the patient cannot see very well what is being done. An empty spectacle frame capable of holding lenses is placed upon the patient's face, and she is told to read illuminated test type in the corner of this darkened room; then, both eyes being open, a strong convex lens is placed in front of one of them so as to obscure its vision, and she is told to read again; then the other eye is treated in the same way. She does not know at the time that this glass obscures the vision, and this manœuvre is generally sufficient to confuse the patient to such a degree that she will read all the letters with either eye, not being quite sure with which eye she is actually reading. Having thus proved to our satisfaction that the patient can read with either eye, we resort to Snellen's coloured test-types, which are red and green, also placing a red and green glass in front of each eye respectively, which isolates the eyes, so that one eye can only read red and the other only green letters. Now she is told to read, and she will at once read both the red and green letters, showing that both eyes are working, and that she has been mistaken about her sight. The condition is, no doubt, an early phase of hysteria, although very different from actual hysterical amblyopia. Older women also come with the same complaint. In men the motive is often more apparent, but even then they fall into the same trap by aid of the mystifying process above described, and by the time we come to the coloured types they will read the red and green letters indiscriminately.

